# Thermal properties and mechanical behavior of hot pressed PEEK/graphite thin film laminate composites

**DOI:** 10.1038/s41598-023-39905-w

**Published:** 2023-08-07

**Authors:** Bakytzhan Sariyev, Alina Abdikadyr, Temirlan Baitikenov, Yerbolat Anuarbekov, Boris Golman, Christos Spitas

**Affiliations:** 1 Department of Mechanical and Aerospace Engineering, School of Engineering and Digital Sciences, Nazarbayev University, 010000 Astana, Kazakhstan; 2 Department of Chemical and Materials Engineering, School of Engineering and Digital Sciences, Nazarbayev University, 010000 Astana, Kazakhstan

**Keywords:** Engineering, Materials science

## Abstract

This work studies high-performance laminate composite materials made of graphite and poly(ether-ether-ketone) (PEEK). The main objective was to enhance graphite's inherent properties by the addition of PEEK to produce materials with improved thermal and mechanical stability for high-performance applications. The composites were fabricated using a hot press method at a temperature below 310 °C. The newly formed materials were then subjected to various tests, including Scanning Electron Microscopy, Thermogravimetric Analysis, mechanical properties tests, nanoindentation tests, and X-Ray Diffraction to assess their structural, thermal, and mechanical properties. Our findings showed a substantial interfacial interaction between PEEK and graphite, indicating successful composite formation. Both three-layered PEEK/graphite/PEEK (PGP) and five-layered PEEK/graphite/PEEK/graphite/PEEK (PG)_2_P composites exhibited superior thermal stability at high temperatures compared to neat PEEK. Moreover, our mechanical tests demonstrated a 172% increase in ultimate tensile strength of PGP compared to neat graphite. Additionally, nanoindentation tests confirmed an increase in both Young's modulus and hardness of composites. Furthermore, XRD analysis revealed a 35.5% increase in crystallinity in the fabricated composites compared to pristine PEEK. These findings significantly contribute to the field of high-performance composite materials, confirming that the hot pressing of PEEK and graphite sheets results in enhanced thermal and mechanical properties.

## Introduction

Nowadays, carbon-polymer composites find applications in various industries due to their outstanding strength-to-weight ratio. One application of this type of material lies within the aerospace industry, where composites are required to demonstrate exceptional performance in terms of high stress, fracture resistance, and damage tolerance, all while maintaining a significantly reduced weight to enable efficient transport of payloads^1,2^. The usage of carbon-polymer composites can save up to 20% and 40% of weight for primary and secondary structures, respectively. Polymer composites are also versatile in manufacturing, as they can be molded into complex shapes.

PEEK is a thermoplastic material already recommended for use in the aerospace industry. Its great thermal resistance, excellent mechanical performance, and low density compared to metals make it a prospective aerospace material^3^. In the production of PEEK/carbon composites, various sintering processes can be employed, with hot pressing, hot isostatic pressing and sinter forging being the most commonly used methods. Alternatively, the melt blending approach involves melting PEEK particles and subsequently mixing the molten PEEK with fillers. Injection molding is another technique where the molten PEEK/carbon composite is injected into a mold to achieve the desired shape^4–8^. However, nanofillers like CNTs and GNPs have a tendency to form agglomerates, making the homogeneous dispersion of carbon a challenging task^9,10^. Moreover, nanocomposites with GNP and CNT tend to suffer stress defects due to the sharp edges of nanoplatelets and void regions in the microstructure^10,11^. In addition, GNP and CNT have low-cost efficiency in mass production, while inexpensive nanoclays cannot conduct electricity^5^. Furthermore, as many researchers mentioned, cost-effective GNP and GO have sophisticated preparation methods compared with graphite due to additional synthesis steps^11,12^.

In contrast to the fillers, graphite is easier to fabricate, and the production of composites using graphite is more cost-effective and feasible. Another reason for incorporating graphite and polymers in composite materials is their high performance in aerospace and vehicle structures. High-modulus graphite is widely used in missile and space launch vehicle structures. The selection of the reinforcing material has a significant impact on the mechanical, electrical, and thermal performance of the fabricated structures. Four-direction carbon fiber composites are more resistant to chemical ablation than their polymer matrix^13^. Among all carbon group materials, graphite has a layered structure consisting of stacked graphene sheets that allow for a delocalized electron sea, making it a better electrical conductor than carbon nanotubes and CNT^14^. The layered structure of graphite contributes to its superior thermal properties^15^. Based on the aforementioned advantages, graphite was chosen as the appropriate material for composite preparation in this study.

Many researchers have focused on reinforcing polymer matrices using carbon fillers by melt blending and injection molding. However, both methods used for mass production have limitations, such as the high cost of equipment and materials involved^16^. In contrast, the hot pressing method offers a cost-effective, viable, precise, and expeditious alternative to conventional techniques like melt blending and injection molding. By subjecting the composite materials to elevated temperatures and pressures, this approach facilitates the creation of a single, strong structure with the desired shape and dimensions. The majority of studies conducted thus far have centered around the utilization of the hot press method for fabricating powder-based PEEK/graphite composites^16–18^. However, to the best of our knowledge, no investigations have been reported regarding the production of multi-layered PEEK/graphite laminate composites using the hot press method.

The anisotropic nature of PEEK/graphite composites allows for a significant level of electrical conductivity facilitated by the graphite sheets. In contrast, PEEK acts as an insulator, directing the flow of current primarily across the graphite layers. Such a composite could have a vast application, for example, in an electrical bus working in a temperature range between 0 and 80 °C in space satellite antennas. In addition, a superior strength-to-weight ratio and high electrical conductivity of PEEK/graphite composites make them well-suited as structural elements in multi-layered membranes and sandwich panels, eliminating the need for aluminum structural supports. Other possible PEEK/graphite composite applications are EM and X-ray radiation shielding for space satellites. Several studies have demonstrated that PEEK exhibits excellent resistance to X-ray and gamma radiation^19,20^.

In this work, we focused on the fabrication of PEEK/graphite composites using the hot press method. We examined the mechanical and thermal properties by tensile test, nanoindentation, and TGA. In addition, the crystallinity behavior of the PGP composite was studied.

## Experimental

### Materials

PEEK APTIV 1000 polymer was purchased from Goodfellow Cambridge Ltd. in the form of a thin sheet of thickness 8 μm with a density of 1.3 g/cm^3^. The graphite sheets 8″ × 8″ with a thickness of 25 μm and a density of 2 g/cm^3^ were purchased from Graphene Supermarket (USA). Table 1 compares the physical properties of PEEK and graphite, utilizing data provided by the manufacturer.

**Table 1:** Physical properties of PEEK and graphite.

Property	PEEK	Graphite
Density, g/cm^3^	1.3	2
Melting temperature (°C)	343	3600
Tensile strength (MPa)	70	16.8
Thickness (μm)	8	25

### Composite preparation

**Figure 1:**
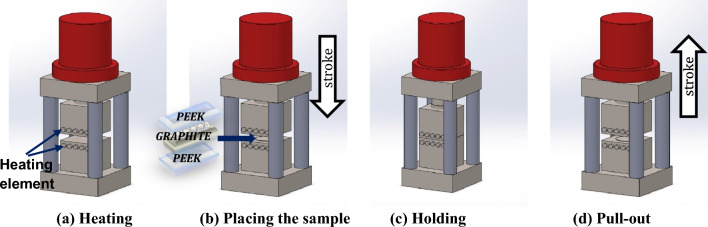
Schematic illustration of hot pressing method.

**Figure 2:**
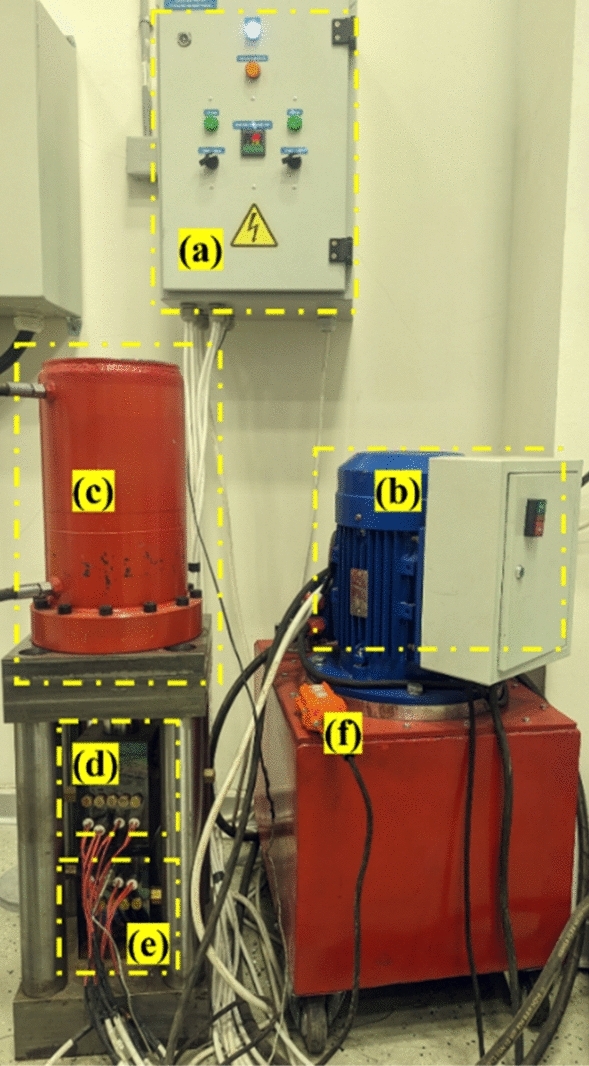
Hot pressing setup: (**a**) control system, (**b**) pump, (**c**) punch, (**d**) upper bulk, (**e**) lower bulk, and (**f**) control console.

(PG)_n_P composites were prepared by the hot pressing method, as illustrated in Fig. 1. The associated equipment setup is shown in Fig. 2. Aluminum foil was employed on both the bottom and top surfaces of the press to prevent adhesion between the heating plates and the polymer. First, the stainless-steel bulk was heated to 330 °C, close to the melting temperature of PEEK. In-situ thermocouples measured the temperature. After that, a multilayer stack of thin films (PG)_n_P was rapidly introduced, and 400 bar pressure was applied for 10 min to form a laminate. Then, the laminate was maintained under pressure and naturally cooled to the below-glass transition temperature of PEEK. Two different laminates, PGP and (PG)_2_P, of size 100 × 100 mm^2^ were prepared. The weight and corresponding volume fractions of graphite in (PG)nP composites are presented in Table 2. They were calculated based on the thickness of the graphite and PEEK sheets.

**Table 2:** Weight and volume fraction of graphite in composites.

Sample	Thickness (μm)	Graphite volume ratio (%)	Graphite weight ratio (%)
PGP	41	60.9	70.6
(PG)_2_P	74	67.5	76.2

### Characterization methods

**Figure 3:**
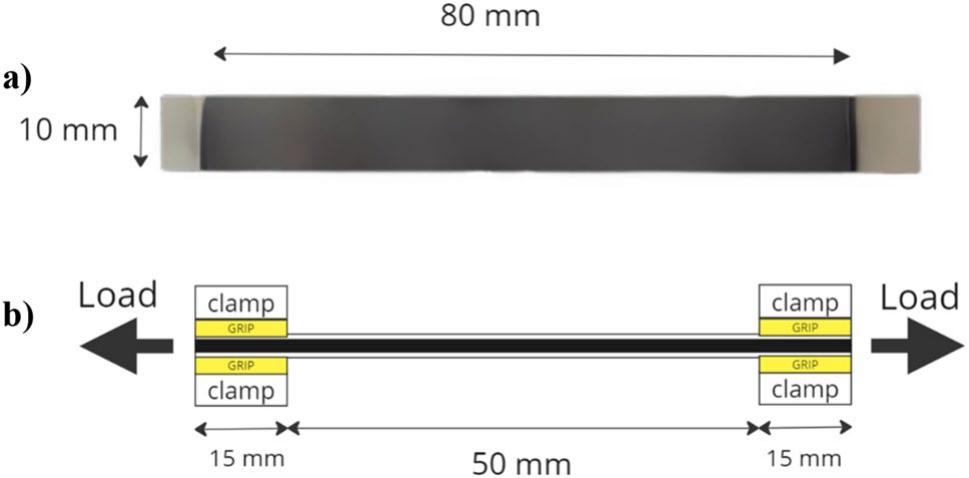
Schematic diagram of (**a**) PGP sample; (**b**) tensile test.

The tensile tests of the fabricated specimens, PGP and (PG)_2_P, were performed using a standard universal test machine (Tinius Olsen 10ST). The samples were clamped with grips at both ends and pulled out with a test speed of 0.1 mm/s, as shown in Fig. 3. A displacement was applied to each test piece until the sample failed. The tensile strength, elongation at break, and Young's modulus were obtained directly from the stress–strain curves. Five samples with gauge dimensions of 80 mm in length and 10 mm in width were tested following ASTM D822 standard for each graphite, PEEK, PGP, (PG)_2_P sample.

Nanomechanical analysis was performed using a Hysitron TI Premier Nanoindenter (Bruker). Flat sheet composites were tested. For each specimen, 20 nanoindentations were made with maximum applied force of 10 mN. The 100 nm diamond-tip Berkovich indenter was used to perform the tests. Specialized software was employed to calculate the hardness and modulus of elasticity of the specimens using the Oliver-Pharr method^21,22^. All nanoindentation tests were performed at a constant temperature of 20 °C.

Furthermore, the morphology of fabricated samples was examined using SEM with integrated EDS (Crossbeam 540, Zeiss). It was operated at 15 kV in the secondary electron mode. A thin gold coating was applied to the specimens to obtain a conductive surface for further SEM and EDS investigations.

XRD tests were conducted at room temperature using a SmartLab SE X-ray diffractometer (Rigaku) with CuKλ radiation (λ = 0.154 nm). XRD data were obtained from the fractured samples of PGP, PEEK, and graphite film samples. The total scanning time was 90 min, the X-ray tube voltage was 45 kV, and the current was 40 mA.

To examine the decomposition temperature range of the PEEK material, TGA measurements were performed using a STA 6000 (Perkin Elmer) under nitrogen atmosphere at a heating rate of 10 °C/min. The sample was heated from 30 to 900 °C, followed by an isothermal stage for 90 min.

## Results and discussion

### Microstructure characterization

**Figure 4:**
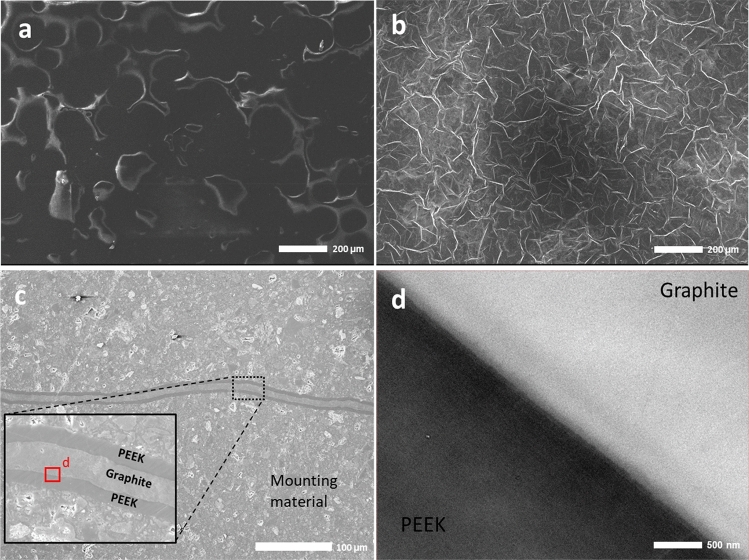
SEM images of (**a**) surface of neat PEEK, (**b**) surface of graphite, (**c**) cross-section of (PG)P, and (**d**) TEM image of PGP interface.

SEM is employed to investigate the surface topography of PEEK and graphite, as well as to observe the cross-sectional interface at a relatively high resolution. This technique helps in understanding the bonding behavior between graphite and PEEK layers, as well as identifying any evidence of cracks or voids. Furthermore, TEM was used to conduct a detailed investigation of the interface. Cross-section analysis revealed a close interfacial adhesion along the whole joint due to the high joining load and polymer melting (Fig. 4). Notably, a solid bond was achieved without the need for material surface functionalization^23–26^.

### Thermal characterization

The thermal stability of the composites was studied using TGA under nitrogen atmosphere. Figure 4 shows PGP, (PG)_2_P, PEEK, and graphite degradation curves. The residue weight loss of neat PEEK at 900 °C varies from 40 to 45%. The decomposition of PEEK and its composites occurs in two steps. The first step takes place at 550–570 °C. Within this temperature range, degradation is attributed to the breakdown of polymer chains containing ketone and ether bonds. The resulting products include carbon dioxide, water, and phenol groups. The second degradation occurs at a temperature above 600 °C. During this step, the residue is cracked and dehydrogenated, leaving thermally stable carbonaceous char^27^.

The addition of graphite slightly increased the degradation temperature from 550 to 570 °C and had no significant effect on the thermal stability. Residue weight of PGP and (PG)_2_P at 900 °C was around 80% and 82% of the initial mass, respectively.

**Figure 5:**
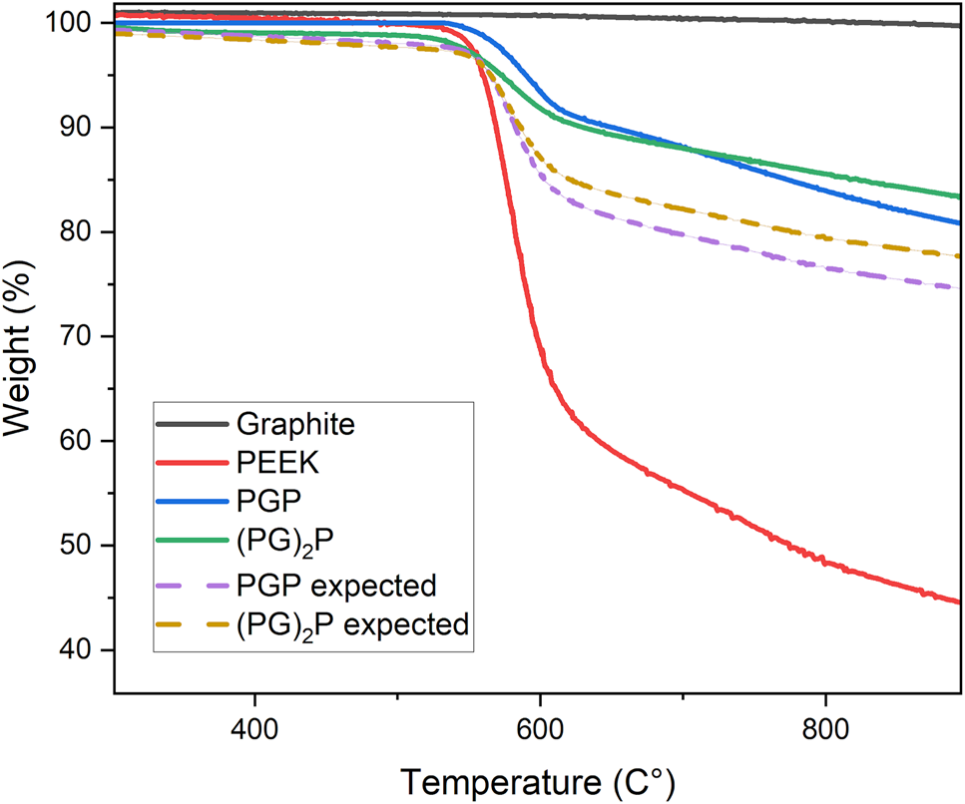
TGA curves of neat PEEK, graphite, PGP, and (PG)_2_P.

A Rule-of-Mixtures graph is plotted in Fig. 5, where the weight percentage of samples and neat materials decreases due to thermal degradation. The chart illustrates that composite performance lies between the curves for neat PEEK and graphite. For samples with higher graphite volume ratio, the weight loss is less due to the better thermal resistance of graphite under higher temperature, according to the rule-of-mixtures. Experimental data showed a higher residual weight percentage than expected, which can be explained by changes in the microstructure of the samples due to the crystallization processes.

### XRD analysis

**Figure 6:**
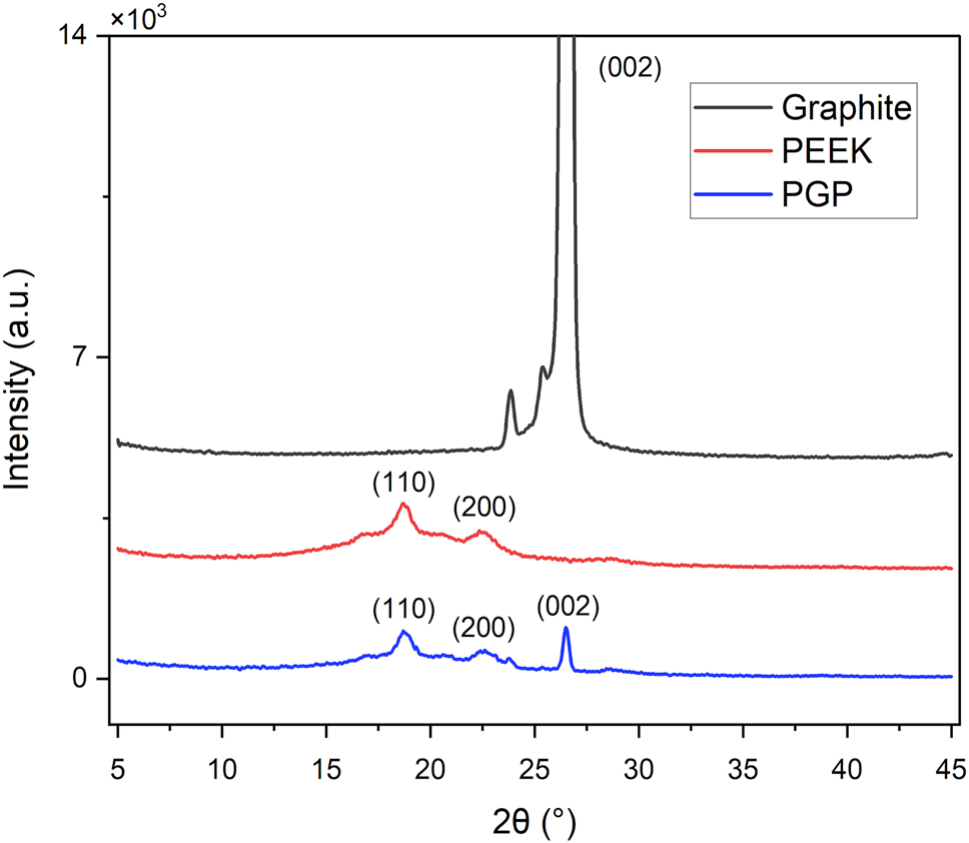
XRD patterns for neat PEEK, graphite, and PGP.

Semicrystalline and amorphous XRD diffraction patterns of neat PEEK, graphite, and PGP over the 2θ range from 5 to 45 °C are illustrated in Fig. 6. Graphite exhibits a (002) sharp peak at 2θ = 24°, indicating its graphitic structure^28^. In addition, the neat PEEK displays peaks at 2θ = 18.82°, 2θ = 20.56°, 2θ = 22.29°, and 2θ = 28.68° belonging to the 110, 111, 200, and 211 planes of the orthorhombic PEEK structure, respectively. The XRD pattern of the hot pressed sample also exhibits a sharp peak at the same angle of 2θ = 26.55° as graphite, indicating the onset of crystallization^29^.

Crystallinity in polymer composites like PEEK plays a vital role, particularly in mechanical and thermal applications. In mechanical applications, such as aerospace and automotive components, as well as biomedical devices, a higher degree of crystallinity tends to result in improved tensile strength, stiffness, and dimensional stability. Moreover, in thermal applications like electronics and energy storage devices, the degree of crystallinity has a significant impact on the material's thermal conductivity and thermal expansion behavior.

Figure 6 shows that the graphite residues remained on a fractured sample after hot pressing, forming a (002) peak with lower intensity. Recent literature^30^ confirms that the broad peaks observed are associated with the amorphous phase, while the sharp peaks correspond to the crystalline phase. Therefore, the crystallinity index, a quantitative indicator of crystallinity for PEEK, PGP, and graphite, was calculated by comparing the integrated peak areas in the 2θ angle range:

1 $$CrI=\frac{{A}_{1}}{{A}_{2}},$$

where A_1_ is the area of all crystalline peaks and A_2_ is the area of all crystalline and amorphous peaks.

From the calculated data, initially, graphite was 67.72% crystalline, while neat PEEK was 28.83% crystalline. After the hot pressing, the crystallinity index of PGP composite accounted for 35.53%.

The obtained results strongly indicate an enhancement of crystallinity of the fabricated samples with the addition of graphite into the PEEK, and similar results were reported by Batakliev et al.^31^. In addition, Harris et al.^32^ investigated the crystallization behavior of neat PEEK with carbon-containing filler added to the polymer matrix. As a result, graphite acts as a stress initiator for nucleation and increases crystallization within the polymer at temperatures close to the melting temperature of PEEK^32^.

### Mechanical properties

#### Tensile test

**Figure 7:**
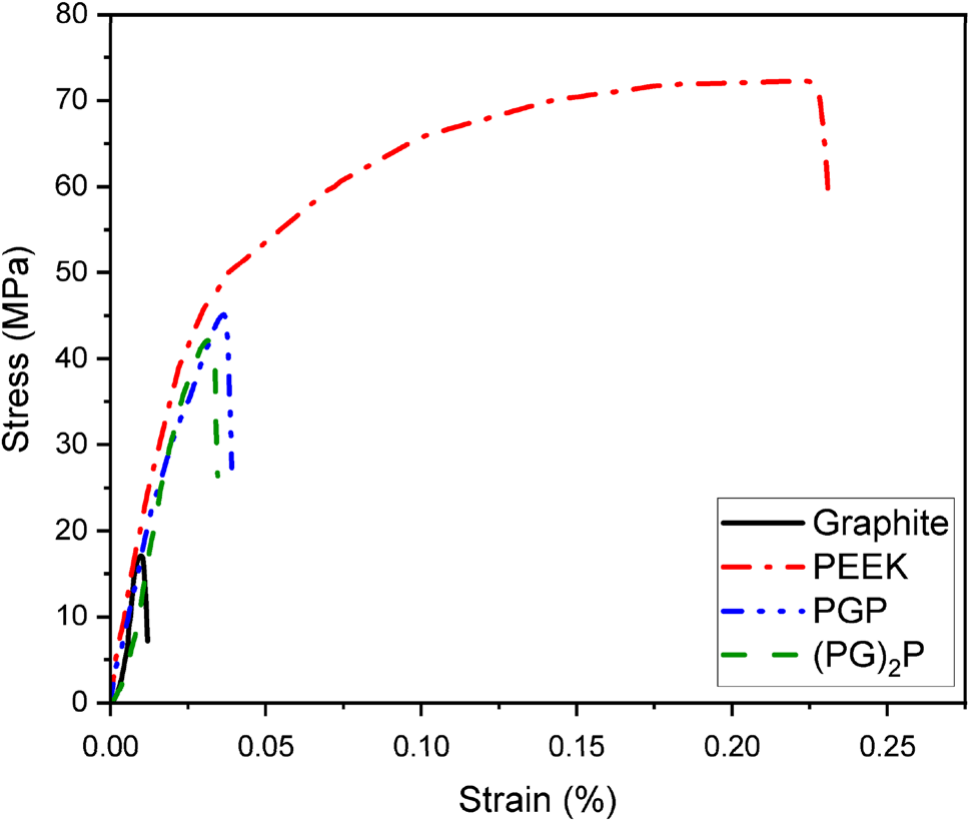
Tensile curves for neat graphite, PEEK, PGP, and (PG)_2_P.

The tensile tests examined the effect of PEEK on the mechanical properties of graphite, and the resultant graphs for neat graphite, PEEK, PGP, and (PG)_2_P are shown in Fig. 7.

Fracture toughness is assessed by evaluating the area under the stress–strain curve. The extent of this area directly correlates with the ductility of the composite. Among the four composites analyzed, the graphite composite exhibits the smallest area, indicating greater brittleness. On the other hand, (PG)_2_P composite displays a larger area, suggesting improved ductility and reduced brittleness. Thus, PEEK makes the composite more ductile^26^. The results demonstrated a six-fold increase in elongation prior to fracture, progressing from graphite to PGP. The inclusion of PEEK results in a reduction in the stiffness of composites (Table 3). To calculate the Young's modulus of the composites from the stress–strain graph, specific points were chosen on each curve that corresponded to strain values of 0.011% and 0.021%. The slopes of the lines passing through these points were then utilized to determine the stiffness of the composites.

**Table 3:** Tensile properties of neat graphite, PEEK, PGP and (PG)_2_P.

Samples	E (GPa)	UTS (MPa)	Elongation (mm)
Graphite	4.33	16.8	0.6
PEEK	1.63	71.2	12.3
PGP	1.85	45.7	4.2
(PG)_2_P	2.1	42.8	1.8

The results indicated that (PG)_2_P exhibited a 48.5% decrease in Young's modulus, while PGP showed a 42.7% decrease compared to neat graphite. A reduction in modulus can be explained by the low modulus of PEEK and its moderate ratio in the fabricated samples, which leads to the high interface with graphite laminate. Similar trends in the modulus of PEEK graphite composites have been reported in studies involving melt-blending^28^. Additionally, the hot press method employed at high pressure facilitated strong adhesion between the layers of the composite. As a result, efficient load transfer was observed between the graphite and PEEK layers.

The volume percentages of PEEK in PGP and (PG)_2_P composites were determined by considering the thickness of graphite and PEEK sheets in the fabricated composites. (PG)_2_P and PGP showed approximately 154.7% and 172% increases in UTS regarding graphite (Table 3). This increase can be attributed to the volume ratio of PEEK, 39% and 32.5% for PGP and (PG)_2_P, respectively. Based on our tensile testing results, the ultimate tensile strength (UTS) of graphite was determined to be 16.8 MPa, while pure PEEK exhibited a UTS of 71.2 MPa. Therefore, it can be concluded that the UTS of the composite cannot exceed that of PEEK and should not be lower than the UTS of graphite.

In fabricated composites, the load transfer strengthens, and the ultimate tensile stress increases due to the interfacial interaction between PEEK and graphite laminates. Although the strength of PGP and (PG)_2_P composites lies between that of pristine graphite and pristine PEEK, it is worth noting that the crystalline form of PEEK demonstrates a higher UTS compared to neat PEEK. When samples subjected to the tensile tension in a parallel setup, more loads will be distributed to the element with the highest stiffness. In both PGP and (PG)_2_P composites, the graphite sheet stiffness is higher than PEEKs; as a result, more of the load will go to the graphite. It was observed that the breaking point primarily occurred in the middle region of the specimen, away from the clamping ends. During these tests, the deviation in the thickness of the specimens was found to be almost negligible, ensuring consistent results.

The Force-to-Weight Ratio is a metric used to determine the maximum force that specimens can withstand relative to their weight. This ratio is calculated using data obtained from the results of a tensile test and the weight of the specimens. PGP has a FWR almost 3.3 times higher than the graphite, and (PG)_2_P exceeds graphite's results by three times (Table 4). These findings indicates that the composite materials are stronger than graphite alone, and with the same weight, they can withstand a higher force before experiencing breaking.

**Table 4:** Force-to-weight ratio of neat graphite, PEEK and PEEK/graphite composites.

Samples	FWR (N/μg)
PEEK	0.556
PGP	0.247
(PG)_2_P	0.224
Graphite	0.074

### Nanoindentation

The hardness and Young's modulus of PEEK, PGP, and (PG)_2_P were measured using a nanoindentation technique, which has been shown to be effective in measuring the surface mechanical properties of thin films^33^. Load–displacement curves were obtained from 20 nanoindentations of three samples at a maximum load of 5 mN. The mechanical properties of samples were calculated using the Oliver-Pharr method based on the nanoindentation test results^21^. Hardness was calculated as follows^22^:

2 $$H{ }\left( {{\text{mean}}\,{\text{contact}}\,{\text{pressure}}} \right) = \frac{{P{ }\left( {{\text{inner}}\,{\text{load}}} \right)}}{{A \left( {{\text{projected}}\,{\text{contact}}\,{\text{area}}} \right)}}.$$

**Figure 8:**
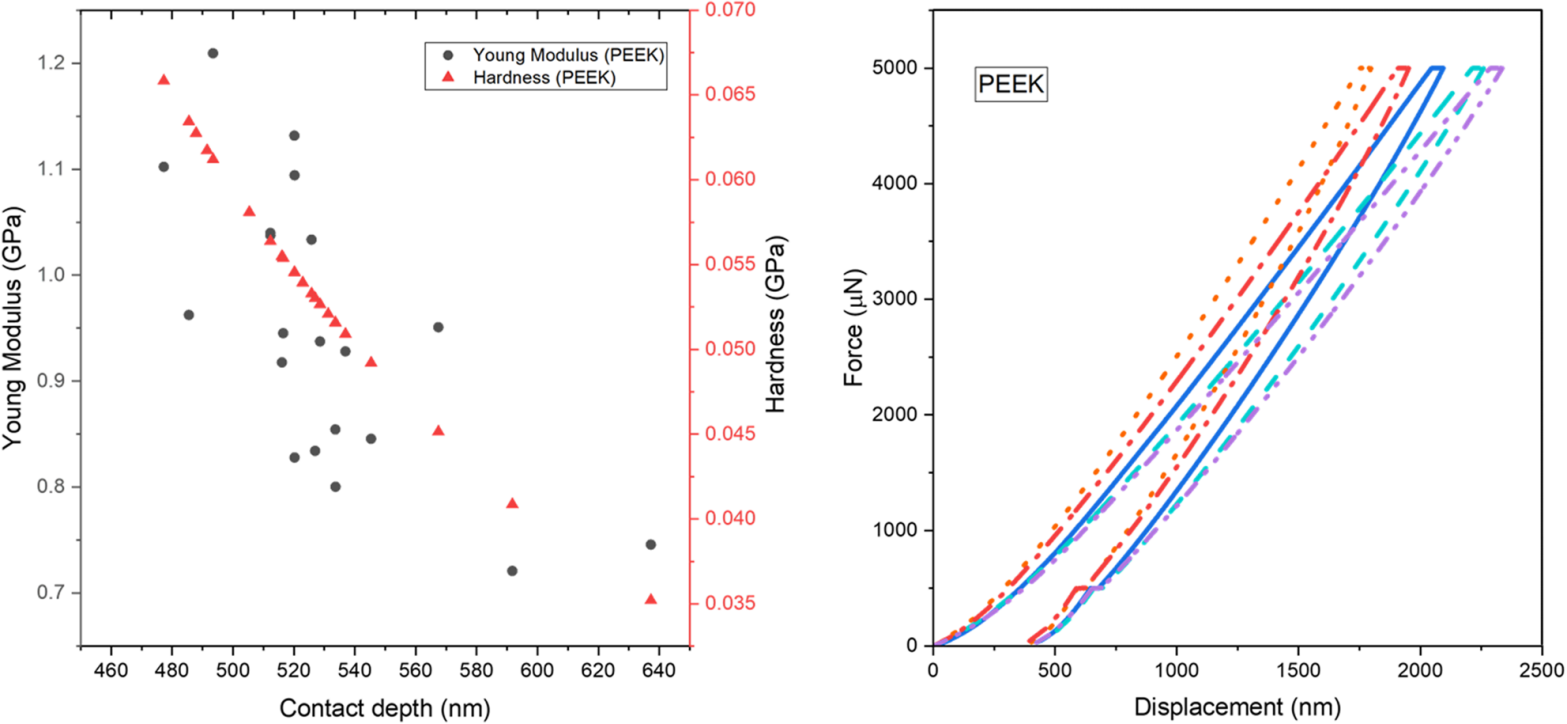
(**a**) Young modulus, hardness of PEEK, (**b**) load–displacement curves of PEEK.

**Figure 9:**
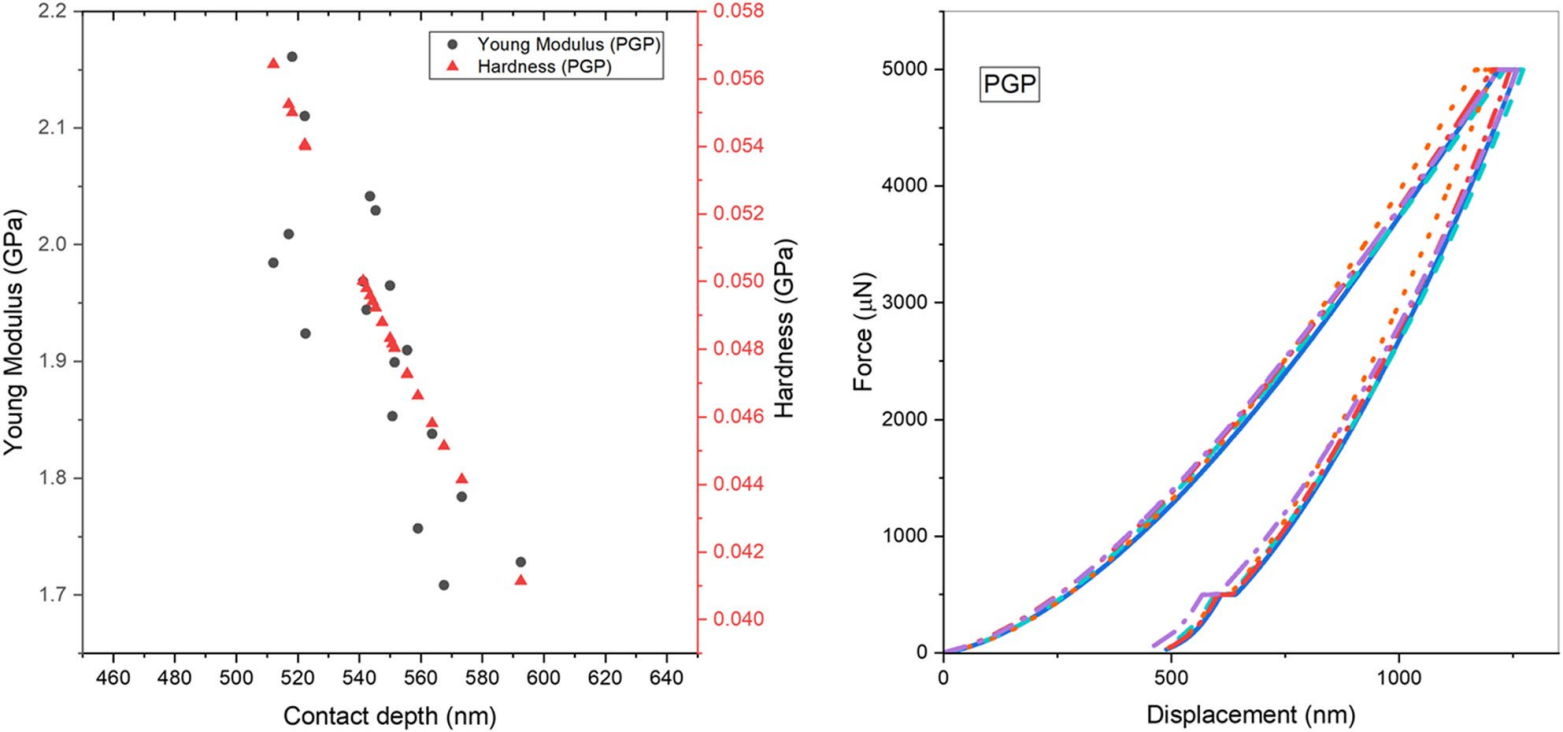
(**a**) Young modulus, hardness of PGP, (**b**) load–displacement curves of PGP.

**Figure 10:**
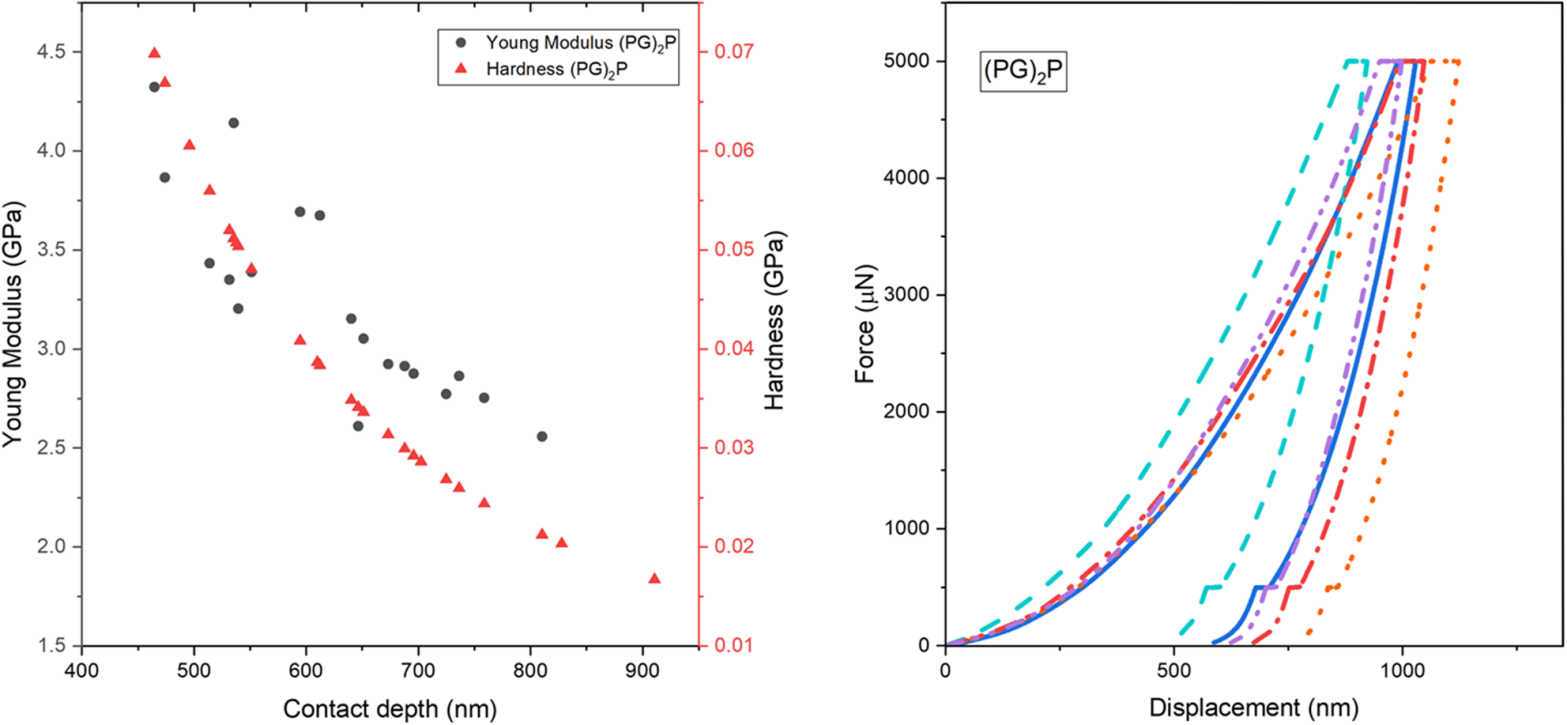
(**a**) Young modulus, hardness of (PG)_2_P, (**b**) load–displacement curves of (PG)_2_P.

Figures 8, 9 and 10 depict typical load–displacement curves of PEEK, PGP and (PG)_2_P respectively. A slight scatter can be observed in the measured Young's modulus profiles due to the friction between the indenter and samples. The depth values in the load-depth curves correspond to the extent of indenter penetration into the material during the nanoindentation test. The observed shift towards lower depth values in the case of (PG)_2_P indicates that this composite exhibit greater resistance to indentation compared to PGP and PEEK. This shift can be attributed to the increased number of graphite layers in the fabricated composites. Thus, the values above the penetration depth of 820 nm and below 520 nm were not considered to avoid an error during the indentation tests^33^.

Table 5 summarizes the values of hardness and Young's modulus for three thin films: PEEK, PGP, and (PG)_2_P. An average value of 20 indentations with their standard deviation is depicted for each sample. The results show that (PG)_2_P has a higher value for both elasticity and hardness modulus than PGP and PEEK. Thus, it can be concluded that increasing the volume of graphite in the composite makes the material stiffer. Also, PEEK's lowest modulus of elasticity and hardness demonstrated that it is more ductile than composites In the previous study conducted by Puértolas et al.^30^, it was concluded that the addition of GNP enhances the surface hardness and tribological properties of PEEK-based materials. Their results obtained from tensile and nanoindentation tests indicated an increase in Young's Modulus, a decrease in tensile strength, and a slight increase in hardness for the GNP/PEEK composite.

**Table 5:** Nanoindentation hardness and Young’s modulus of all tested thin films.

Sample	Average hardness (GPa)	Average Young’s modulus (GPa)
PEEK	$0.054\pm 0.007$	$0.924\pm 0.128$
PGP	$0.164\pm 0.025$	$1.640\pm 0.159$
(PG)_2_P	$0.178\pm 0.045$	$2.580\pm 0.427$

Based on the results of the nanoindentation test, it was observed that (PG)_2_P possesses superior surface mechanical properties compared to PEEK. Specifically, (PG)_2_P exhibited a modulus of elasticity three times higher than that of PEEK.

In summary, the moderate difference in results between the tensile test and nanoindentation technique can be attributed to the different measurement approaches. The tensile test measures the overall effective modulus under dynamic conditions reflecting the bulk mechanical properties of the sample, while nanoindentation measures the surface mechanical properties. The results obtained by nanoindentation are generally higher in modulus of elasticity and hardness compared to the tensile test due to the influence of surface tension as well as factors such as size effect, non-linear viscoelasticity, particle density, strain exceeding the elastic limit, and imposed hydrostatic stress values^34^.

#### Fracture analysis

**Figure 11:**
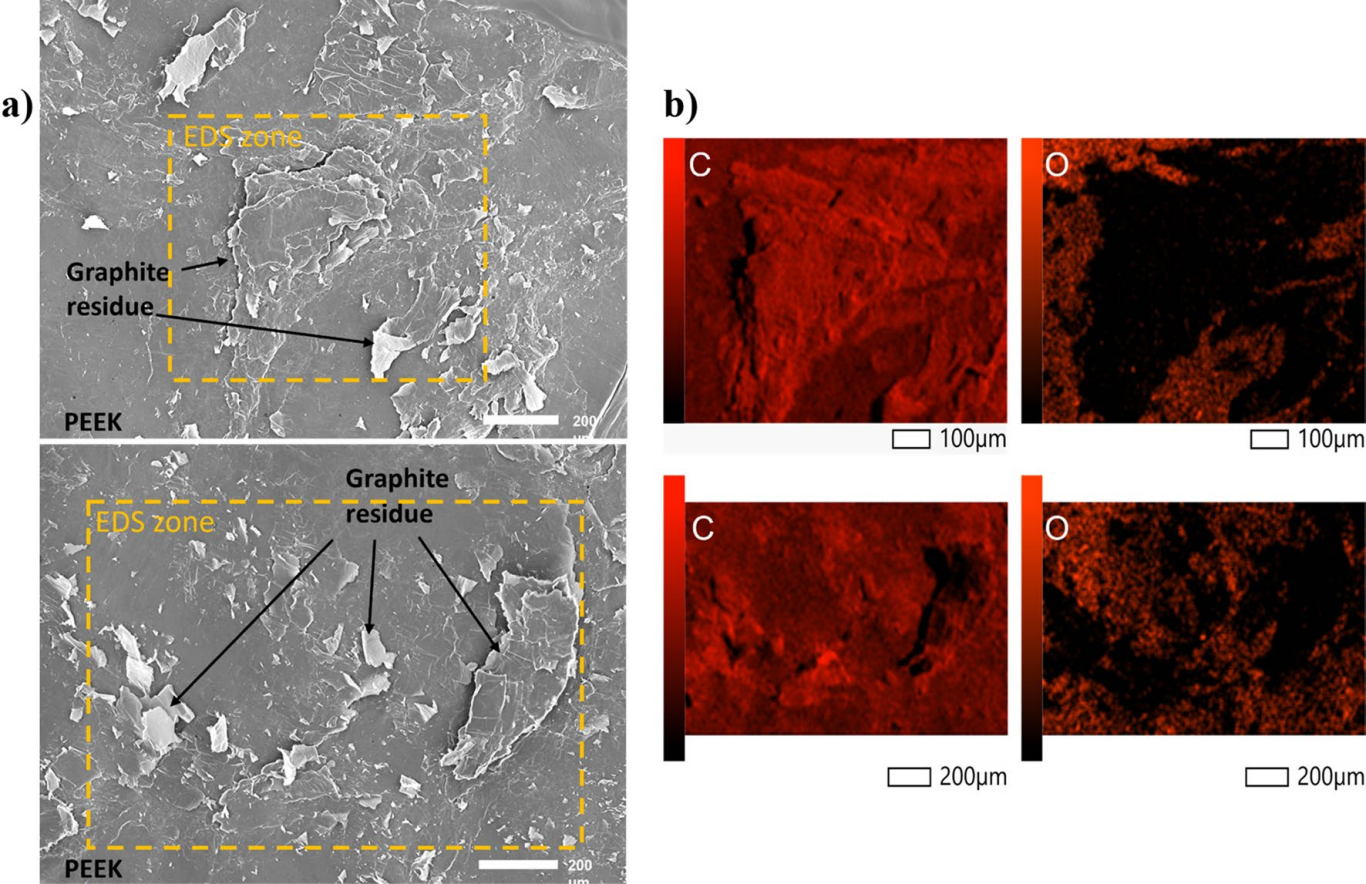
(**a**) SEM and (**b**) EDS images of the fractured surfaces of PGP specimen.

The residual material on PEEK was determined by examining the microstructures of fracture surfaces after tensile tests using SEM and EDS (Fig. 11). Through the combined use of SEM and EDS, we were able to observe and confirm the presence of graphite flakes on the fractured surface. The results of the EDS analysis revealed that there was no oxygen and a high carbon content, indicating presence of graphite flakes from bond formation between two different materials during the hot press process. Graphite, being more brittle than PEEK, exhibits fracture characteristics that involve brittle fracture with angular edges and flat surfaces. The observation of such features in the SEM images suggests the fracture process partially occurred within the graphite flakes. The formed flakes are likely responsible for strengthening the composites and improving their mechanical properties. The exact bonding mechanism between PEEK and graphite will be a topic for further investigation in future research.

## Conclusion

In this study, high-performance PEEK/graphite thin film laminate composites were fabricated by the hot pressing method. This approach significantly improved the thermal and mechanical properties of the composites, as observed through a series of comprehensive tests and analyses. Our investigations showed a significant interfacial interaction between PEEK and graphite, indicating successful formation of the composite. SEM images provided evidence of this interaction, with a visible close contact between the graphite and PEEK layers. Thermogravimetric analysis showed an improvement in thermal stability with the introduction of graphite. The (PG)_2_P samples maintained an 80% weight ratio at 900 °C, nearly twice that of pristine PEEK. Furthermore, the crystallinity index of the fabricated composite, as evaluated by XRD analysis, increased from 28.83 to 35.5% compared with pristine PEEK. Mechanical tests revealed a remarkable enhancement in the UTS in the PEEK/graphite laminate, with PGP showing a 172% increase compared to neat graphite. Nanoindentation tests confirmed a notable increase in both Young's modulus and hardness, as the surface mechanical properties of (PG)_2_P exhibited values three times higher than those of neat PEEK. Additionally, the force-to-weight ratio of PGP and (PG)_2_P increased 3.3 and 3 times compared to pristine graphite, respectively.

These findings underline the promise of our fabrication approach for creating highly thermally stable and mechanically robust composites. The strategy of preparing PEEK/graphite laminates through hot pressing offers a viable pathway for the production of advanced materials for high-performance applications. The substantial improvements in both thermal and mechanical properties observed in this study underscore the potential of these composites for wide-ranging applications.

## Data Availability

All data generated or analyzed during this study are included in this published article.

## Abbreviations

CNT: Carbon nanotubes
EM: Electromagnetic
EDS: Energy dispersive X-ray spectroscopy
FWR: Force-to-weight ratio
GNP: Graphene nanoplatelets
GO: Graphene oxide
PEEK: Poly(ether-ether-ketone)
PGP: PEEK-graphite-PEEK
(PG)_2_P: PEEK-graphite-PEEK-graphite-PEEK
SEM: Scanning electron microscope
STA: Simultaneous thermal analyzer
TEM: Transmission electron microscopy
UTS: Ultimate tensile strength
XRD: X-ray diffraction
